# Lateral Habenula Beyond Avoidance: Roles in Stress, Memory, and Decision-Making With Implications for Psychiatric Disorders

**DOI:** 10.3389/fnsys.2022.826475

**Published:** 2022-03-03

**Authors:** Phillip M. Baker, Victor Mathis, Lucas Lecourtier, Sarah C. Simmons, Fereshteh S. Nugent, Sierra Hill, Sheri J. Y. Mizumori

**Affiliations:** ^1^Department of Psychology, Seattle Pacific University, Seattle, WA, United States; ^2^CNRS UPR 3212, Institut des Neurosciences Cellulaires et Intégratives, Center National de la Recherche Scientifique, University of Strasbourg, Strasbourg, France; ^3^CNRS, Laboratoire de Neurosciences Cognitives et Adaptatives (LNCA), UMR 7364, Université de Strasbourg, Strasbourg, France; ^4^Department of Pharmacology and Molecular Therapeutics, School of Medicine, Uniformed Services University of the Health Sciences, Bethesda, MD, United States; ^5^Department of Psychology, University of Washington, Seattle, WA, United States

**Keywords:** lateral habenula, memory, reward, motivation, sleep, psychiatric illnesses, early life stress

## Abstract

In this Perspective review, we highlight some of the less explored aspects of lateral habenula (LHb) function in contextual memory, sleep, and behavioral flexibility. We provide evidence that LHb is well-situated to integrate different internal state and multimodal sensory information from memory-, stress-, motivational-, and reward-related circuits essential for both survival and decision making. We further discuss the impact of early life stress (ELS) on LHb function as an example of stress-induced hyperactivity and dysregulation of neuromodulatory systems within the LHb that promote anhedonia and motivational deficits following ELS. We acknowledge that recent technological advancements in manipulation and recording of neural circuits in simplified and well-controlled behavioral paradigms have been invaluable in our understanding of the critical role of LHb in motivation and emotional regulation as well as the involvement of LHb dysfunction in stress-induced psychopathology. However, we also argue that the use of ethologically-relevant behaviors with consideration of complex aspects of decision-making is warranted for future studies of LHb contributions in a wide range of psychiatric illnesses. We conclude this Perspective with some of the outstanding issues for the field to consider where a multi-systems approach is needed to investigate the complex nature of LHb circuitry interactions with environmental stimuli that predisposes psychiatric disorders.

## Introduction

The lateral habenula (LHb) clearly plays a role in learning and memory since LHb disruption produces deficits on tasks that require the processing of contextual information (Baker et al., 2015; Durieux et al., 2020), spatial working memory (Mathis and Lecourtier, 2017; Mathis et al., 2017), and/or stimuli associated with negative valence outcomes (Stamatakis et al., 2016; Knowland and Lim, 2018; Sosa et al., 2021). Across these diverse types of memory and cognitive processing, a fundamental contribution of the LHb may be to constantly monitor one’s current internal state relative to external environmental conditions so that behaviors can be modified as needed (Baker et al., 2015; Mathis and Lecourtier, 2017; Lecca et al., 2020). Such a contribution appears to rely on the integration and signaling of cognitive, motivational/emotional, and behavioral state information (Sutherland, 1982; Chastrette et al., 1991; Nair et al., 2013; Mendoza, 2017; Shepard and Nugent, 2021). For example, LHb responds to positive and negative choice outcomes (Matsumoto and Hikosaka, 2009; Li et al., 2019), the generation of prediction error signals (Hong and Hikosaka, 2013; Tian and Uchida, 2015), changes in motivational and physiological states [e.g., stress, time of day, etc., (Shepard et al., 2018b; Salaberry et al., 2019; Langlois et al., 2021)], and changes in behavioral state (Baker et al., 2015; Nuno-Perez et al., 2018; Lecca et al., 2020).

Functional efferent and afferent connections of the habenula [reviewed in detail in Baker et al. (2015) and Quina et al. (2015)] to areas including the frontal cortical areas (Mathis et al., 2017), the basal ganglia (Wallace et al., 2017), the ventral tegmental area (Stamatakis et al., 2013; Liu et al., 2021). Despite increasing supporting evidence of this broad view of LHb function, a number of significant issues remain to be resolved if we are to sufficiently understand the adaptive relevance of the LHb for everyday memory function. These advances will aid in the development of novel interventions for neuropsychiatric conditions that have been linked to LHb dysfunction such as depression, anxiety, and addiction.

In the following, we focus on key outstanding issues related to two widely held concepts regarding LHb function: 1) The LHb serves as a critical interface for context memory and internal emotional state information, and 2) This integrative role positions the LHb to play a key role in specific psychopathological symptoms due to poor integration of context and emotional information, such as that which occurs when stressed. Evidence to support these general concepts of LHb function is highlighted along with examples of research that exemplify important unresolved issues. It is then suggested that our understanding of the contribution of the LHb to behavior can be substantially enhanced by greater inclusion of more ethologically-relevant tasks. Finally we conclude with suggestions for paths forward.

## Role of the Lateral Habenula in Memory Processes: An Interface Between Context and Internal Emotional State

A growing number of studies have demonstrated, in rodents, that pharmacological or chemogenetic inhibition of LHb induced deficits of several types of memory, including long-term spatial memory in the water maze (Mathis et al., 2015), contextual memory in an object-based recognition task (Goutagny et al., 2013), short-term memory in a delayed non-matching to position task (Mathis and Lecourtier, 2017), fear memory in a trace fear-conditioning paradigm (Durieux et al., 2020) as well as inhibitory avoidance (Tomaiuolo et al., 2014) [see also Song et al. (2017)]. One noteworthy aspect of these examinations is that the engagement of the LHb in learning and memory appears to relate to two aspects of the ongoing situation: its emotional valence and the context in which it occurs.

It does not seem surprising that the LHb is particularly engaged in memory tasks requiring the processing of contextual cues during negative emotional situations, as it has a major role in signaling aversion (Hennigan et al., 2015; Li et al., 2019) and it shows strong activation in response to a large number of stressors (Chastrette et al., 1991; Lecca et al., 2017; Li et al., 2019). In the water maze, LHb dysfunctions not only induced memory deficits, i.e., a greater distance to reach the hidden platform during training and a lower time spent in the target quadrant (i.e., the area where the platform—which has since been withdrawn—was located) during the retention test [see Mathis et al. (2015)], but also led to signs of exacerbated stress, i.e., excessive thigmotaxic behavior (swimming along the edge of the pool) in conjunction with an increased corticosterone (CORT) release [(Mathis et al., 2015, 2018); see also Jacinto et al. (2017)].

These types of results following LHb dysfunction suggest that one of its main roles could be to process different modalities of an ongoing situation, including external environmental cues and internal emotional state, and to participate in the elaboration of appropriate behavioral responses. Hence, the LHb integrates external information as well as physiological, internal, signals. In that regard recent studies showed that the LHb signals stress and punishment in a context-dependent manner, as combination of stressors or contextual illumination reduces LHb stress response (Zhang et al., 2016; Huang et al., 2019). These findings suggest a yet underdetermined influence of external conditions over the LHb functions. Further studies are required to better understand how and in which conditions the LHb can simultaneously deal with external (context, nature of the threat) and internal (CORT levels, circadian rhythm) information. Such a role for the LHb in both both stress- and memory-related information processing raise an important question: are cognitive deficits a primary consequence of LHb dysfunction, secondarily inducing defective stress coping, or is an impossibility to cope with a stressful situation the primary consequence of LHb dysfunction, secondarily inducing learning and memory deficits?

At this point it is hard to answer this question. Indeed, most of the behavioral tests used to assess memory in rodents often include an aversive component to motivate the animals; electrical foot shocks in fear conditioning, cool water to swim in in order to find a hidden platform in the water maze, or food restriction in a variety of tasks using delayed non-matching to position paradigms (although the latter also imply reward-related processes). On the contrary, it might seem simpler to address stress response processes. Hence, as mentioned above, the LHb seems to be a crucial structure engaged in the response to stressors and in signaling aversive situations. The impact of stress over cognitive performances is well described. While low levels of stress can improve performances, a high or prolonged stress will eventually induce deficits (Arnsten, 2015), especially memory deficits (Kim and Diamond, 2002; Roozendaal et al., 2009). A simple hypothesis would be to consider that, if altered, the engagement of the LHb in stress integration will interfere with memory processes, subsequently leading to performance deficits. This would explain why pharmacological inhibition of the LHb during the acquisition phase of each training day prevented learning in a water maze paradigm (Mathis et al., 2015). Such intervention likely increased the stress load across training days, resulting in a flat learning curve. Indeed, impaired rats showed an increased level of thigmotaxic behavior (Mathis et al., 2015), which can be attributed to defective stress coping, and exacerbated CORT levels (Mathis et al., 2018). This is in accordance with the fact that LHb dysfunction induces anxiety-like behaviors on the elevated plus maze (Mathis et al., 2015). However, it might seem contradictory with the fact that when LHb inhibition occurred at the probe test following a drug-free training phase that should have attenuated potential stress responses (during which one can therefore postulate that rats had been used to the stressful aspect of the situation and had been able to deal with it), it nonetheless created retrieval deficits (Mathis et al., 2015). In addition, during this probe test rats showed a reduced swim speed, suggesting a “calm” exploration of the apparatus. We have also found using a different paradigm, that following habituation to the testing condition and drug-free training, LHb inhibition impaired memory of object locations in an open field when one of three objections is moved from a previous location and replaced with a novel object (Goutagny et al., 2013). All together these results suggest that the LHb role in stress processing is not likely the only reason for the observed memory deficits.

These findings appear to support the idea that cognitive deficits are a primary consequence of LHb dysfunctions, secondarily inducing exacerbated stress. Indeed, the thigmotaxic behavior observed in the water maze following LHb inhibition might reflect the engagement of a default behavioral response as a consequence of a lack of knowledge about the platform location. Such a behavior might be interpreted as a “low-cost” strategy triggered when no memory-based strategy is available. The CORT elevation would then be a consequence required for the physical effort and partially reflecting stress.

Finally, a third case would be that the LHb processes stress- and memory-related information in an independent manner. However, as said earlier, the existing paradigms assessing cognitive processes do not necessarily give the possibility to address stress and memory independently and then together. Indeed, the intrinsic aversive aspect of most of the behavioral tests assessing memory prevents from dissociating these two aspects. One possibility though could be to add a supplementary stressor and assess the effect of this other stressors on memory performances.

Beside the behavioral paradigms, understanding how the LHb receives contextual and stress-related information could help to answer this chicken and egg question. Indeed, the LHb position in the central nervous system is of great interest with regard to stress and cognitive processes. The LHb belongs to the dorsal diencephalic conduction system conveying information from the prefrontal cortex, several septal nuclei, the hypothalamus or the entopeduncular nucleus to midbrain monoaminergic areas such as the raphe, ventral tegmental area and the locus coeruleus (Roman et al., 2020).

Understanding how the LHb receives contextual and stressful information would help to answer this chicken and egg question. Interestingly, upon cognitive testing, a functional connectivity between the LHb and both the mPFC (Mathis et al., 2017) and HPC (Baker et al., 2019; Durieux et al., 2020) has been shown to exist. In addition, the LHb and HPC, although not directly anatomically connected, likely communicate whether it is during exploration of an unfamiliar environment or during rapid eye movement (REM) sleep episodes (Aizawa et al., 2013; Goutagny et al., 2013). The link with sleep is of particular interest as communication between the LHb and HPC could be related to past experiences and therefore be part of the mechanisms underlying HPC-dependent learning and memory processes. A specific role of the LHb in sleep-dependent processes seems also in accordance with the fact that the LHb shows circadian oscillatory activity and is implicated in circadian-related behaviors (Guilding et al., 2010; Baño-Otálora and Piggins, 2017; Mendoza, 2017; Huang et al., 2019; Salaberry et al., 2019). A better understanding of the LHb-related network conveying memory-related information would help untangle whether memory deficits are at the origin or the consequences of the observed exacerbated stress response in the different memory tasks aforementioned (e.g., water maze, fear conditioning).

Further investigations are needed to fully understand how the different types of information (contextual vs. stress-related) are integrated by the LHb. This could be performed using behavioral paradigms that include repeated stressful situations, in order to potentially capture habituation processes and coping strategies. It would be interesting, in such paradigms, to investigate the activity of the LHb in conjunction with those of prefrontal cortical, hippocampal, and amygdalar regions, and explore the level of communication between those structures according to the different aspects of the paradigm, including the acute response to the stressful procedure, and the coping mechanisms upon repetition of it. Examinations could also include important stress-related structures which send input to the LHb, such as the hypothalamus (Lecca et al., 2017; Trusel et al., 2019), the entopeduncular nucleus (Stephenson-Jones et al., 2016; Li et al., 2019), frontal cortical areas (Kim and Lee, 2012; Fillinger et al., 2017), and the VTA (Stamatakis et al., 2013) which likely send information related to the emotional valence of the situation, thus positioning the LHb as a cerebral “hub,” linking different macro-systems (Geisler and Trimble, 2008).

It will also be important to better describe the influence of the context over the stress-related aspect of the paradigm. The recent results showing that environmental illumination conditions directly influence the LHb capacity to signal stress through a retino-thalamo-habenular circuit, and participates in the effect of light therapy in depression, is a first step toward this goal (Huang et al., 2019). The recent advances in neuroscience allow *in vivo* circuit specific investigation and will likely participate in elucidating these issues.

## Lateral Habenula Represents a Key Node for Increased Risk of Psychopathology Following Early Life Adversity

It is well-established that exposure to childhood adversity/early life stress (ELS) is a strong predictor for several later life mental disorders, including substance use disorders (SUDs), anxiety and depression (Heim et al., 2010; Lippard and Nemeroff, 2020; Shepard and Nugent, 2020, 2021). Common forms of childhood adversity include child abuse and neglect, domestic violence, and family economic hardship. The recent COVID-19 pandemic shutdowns across the globe have caused detrimental effects on child mental health with the increased risk for domestic violence, child abuse and neglect, compounded by food and housing insecurity (Gotlib et al., 2020; Humphreys et al., 2020; Lawson et al., 2020; Yard et al., 2021). Poor responsivity of psychiatric patients with a prior history of ELS to psychotherapy and/or pharmacotherapy further necessitates a better understanding of the mechanisms and neural circuits that link ELS with mental illnesses to identify potential novel interventional therapeutic targets.

Prominent ELS rodent and primate models employ early disruptions in mother-infant relationship such as a single 24 h maternal deprivation (MD), repeated daily maternal separation (MS), and limited bedding and nesting (LBN) (Macrì et al., 2007; Nishi et al., 2014; Shepard et al., 2018a; Okhuarobo et al., 2020). Although these ELS models may not reflect all types of early adverse experiences, they are associated with persistent depressive-and anhedonia-like behaviors (Tchenio et al., 2017; Authement et al., 2018; Bolton et al., 2018a; Shepard et al., 2018b; Simmons et al., 2020) and altered drug reward (Bolton et al., 2018b; Okhuarobo et al., 2020; Langlois et al., 2021; Levis et al., 2021) suggesting the translational validity of these models for child neglect. However, it should be noted that not all animals that experience ELS develop stress psychopathology or substance use disorders later in life which is also the case for children exposed to adversity (Kalinichev et al., 2002; Moffett et al., 2006; Ordoñes Sanchez et al., 2021). Thus, in preclinical ELS research, differences between predictable (MS) and unpredictable (single prolonged MD and limited bedding and nesting) stressors as well as the duration of separation and alterations in maternal behavior should be taken into account which may confer resistance or vulnerability and directly impact the outcomes in terms of addictive behaviors, depression and mood phenotypes in these models.

Several neural pathways and neurobiological mechanisms such as the hypothalamic-pituitary-adrenal (HPA) axis and extra-hypothalamic corticotropin-releasing factor (CRF) circuits have been identified by which ELS may increase the risk for mood dysregulation, stress-related disorders and addiction (Nemeroff, 2016). Emerging evidence now suggests that ELS-induced alterations of reward- and stress-related brain regions such as ventral tegmental area (VTA), amygdala, nucleus accumbens, prefrontal cortex and LHb may underlie the increased risk for ELS-induced psychopathology (Authement et al., 2015, 2018; Peña et al., 2017, 2019; Tchenio et al., 2017; Bolton et al., 2018a; Shepard et al., 2020; Simmons et al., 2020; Langlois et al., 2021; Oh et al., 2021; Shepard and Nugent, 2021). Specifically, recent studies provided compelling evidence that the LHb is a critical converging brain region for ELS-induced dysregulation of reward circuits (Tchenio et al., 2017; Authement et al., 2018; Bolton et al., 2018b; Simmons et al., 2020). The LHb links forebrain limbic structures with midbrain monoaminergic centers (Schultz, 2010; Cohen et al., 2012; Proulx et al., 2014) and is involved in reward/aversion-related learning and memory processing associated with avoidance from stressful and aversive situations through suppression of dopamine and serotonin systems. Specifically, anatomically and/or functionally diverse neuronal populations within the LHb modulate motivated behaviors through cell type-specific projections to non-overlapping targets including the VTA, substantia nigra compacta, rostromedial tegmental area (RMTg), or raphe nuclei (Stamatakis et al., 2016; Wallace et al., 2017; Cerniauskas et al., 2019; Hu et al., 2020; Lecca et al., 2020). Not surprisingly, LHb dysfunction contributes to a myriad of cognitive, learning, and affective impairments associated with depression, anxiety, psychosis and drug addiction (Graziane et al., 2018; Nuno-Perez et al., 2018; Proulx et al., 2018).

The common finding among studies using ELS models MD (Authement et al., 2018; Shepard et al., 2018b; Simmons et al., 2020; Langlois et al., 2021) and MS (Tchenio et al., 2017) is that ELS promotes LHb hyperexcitability although the underlying mechanisms vary from downregulation of small conductance (SK2) potassium channels and increased protein kinase (PKA) activity in LHb (Authement et al., 2018) to decreased postsynaptic GABA_*B*_R-GIRK signaling arising from entopeduncular nucleus GABAergic inputs to LHb (Tchenio et al., 2017). Additionally, MD in rats persistently increases both tonic and bursting LHb activity from early adolescence to adulthood (Authement et al., 2018; Shepard et al., 2018b; Simmons et al., 2020; Langlois et al., 2021) consistent with the literature that LHb hyperactivity in general (and bursting in particular) contributes to the development of depression-like motivational and social deficits, and anhedonic phenotypes (Yang et al., 2018; Klein et al., 2020). Either chemogenetic inhibition of LHb neurons or deep brain stimulation that reduces LHb activity ameliorates MS-induced depressive-like phenotype in mice (the lack of motivation of mice to avoid an aversive context which is an escapable foot-shock) (Tchenio et al., 2017). Interestingly, juvenile MD rats show an increased active coping behavior in the forced swim test (with an increase in climbing behavior) while late adolescent rats exhibit an increased immobility in the forced swim test, both behavioral phenotypes are reversed by ketamine treatment (Shepard et al., 2018b). More importantly, long-lasting anti-depressant effects of ketamine on MD-induced behavior in young adult rats is associated with a return to normal levels of LHb neuronal excitability (Shepard et al., 2018b). MD also triggers an anhedonic phenotype in natural sucrose reward while also decreasing morphine intake in morphine self-administration acquisition associated with MD-induced glutamatergic plasticity in LHb neurons (Langlois et al., 2021).

Consistently, it has been shown that synaptic transmission from the LHb to the RMTg, a nucleus that suppresses dopamine neuronal activity and signaling, increases during transitions to immobility in the forced swim test to escape this aversive context. Activation of this LHb to RMTg circuit also decreases motivation of rats to work harder to receive sucrose reward in a progressive ratio schedule of operant appetitive task suggesting a critical role for the LHb in regulation of motivation (Proulx et al., 2018). Therefore, it is possible that MD-induced LHb glutamatergic plasticity and LHb hyperactivity could increase the excitatory drive from the LHb to the RMTg and underlie motivational deficits in MD rats. In the future, it is necessary to employ similar circuit-based studies of MD effects on motivation such as progressive ratio schedule in sucrose self-administration, morphine self-administration, or other motivation based effort tasks (see below). Overall, these findings highlight the role of LHb hyperactivity in ELS-induced induction of anhedonic states and altered opioid seeking where limiting LHb activity using novel fast-acting antidepressants such as ketamine or deep brain stimulation could have therapeutic potential. It remains unclear at this point, however, whether these effects are concurrent with broader cognitive and behavioral effects as has been noted with the aforementioned memory related tasks.

**FIGURE 1:**
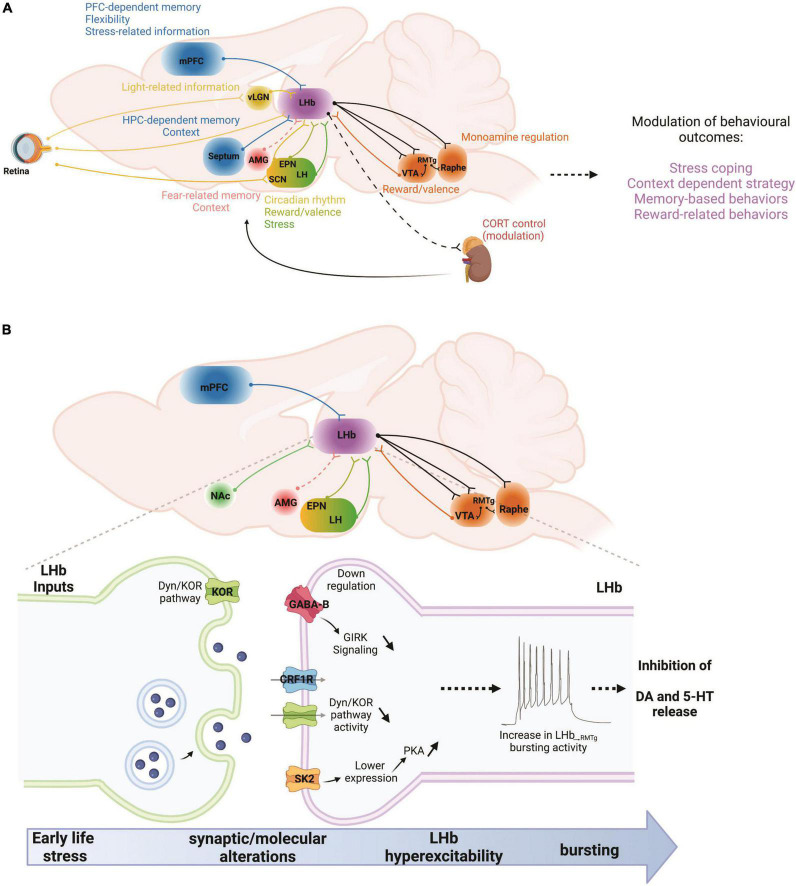
**(A)** In a given situation, the LHb can be viewed as a recipient of multiple types of information stemming from cortical and subcortical regions. Those include memory- and flexibility-related information from the mPFC, or hippocampal memory-related information from the septum. Emotional information arises from the AMG while stress-related information from the mPFC and the LH. Reward and valence-related information reach the LHb from the EPN and the dopamine system. Finally, light-related information reaches the LHb from the retina, either directly or through the vLGN and SCN. Once the LHb has processed this information, they are communicated to the dopamine and serotonin systems, as well as to the HPA axis in order to adapt behavioral responses to changes in environmental constraints. AMG, amygdala; CORT, corticosterone; EPN, entopeduncular nucleus; HPC, hippocampus; LH, lateral hypothalamus; LHb, lateral habenula; mPFC, medial prefrontal cortex; RMTg, rostromedial tegmental nucleus; SCN, suprachiasmatic nucleus; vLGN, ventral lateral geniculate nucleus; VTA, ventral tegmental area. **(B)** Early life stress, such as maternal deprivation (MD) and repeated daily maternal separation (MS) lead to LHb hyperexcitability. Maternal deprivation in particular also increases LHb neuronal bursting and intrinsic excitability. MS-induced LHb hyperactivity is the consequence of altered input communication from at least the EPN with down-regulation of GABABR-GIRK signaling. On the other hand, MD dysregulates Dyn/KOR and CRF-CRFR1 signaling pathways while increasing PKA activity that promotes the downregulation of small conductance (SK2) potassium channels. Increases in LHb bursting and activity can in turn downregulate dopaminergic and serotonergic transmissions through hyperactivation of the RMTg, and therefore promote stress-related disorders such as anxiety and depression. Future studies will require an exploration of the potential contributions of other LHb inputs to such intra-LHb molecular disturbances, for example from the mPFC, NAc, AMG, and LH. AMG, amygdala; EPN, entopeduncular nucleus; LH, lateral hypothalamus; LHb, lateral habenula; mPFC, medial prefrontal cortex; NAc, nucleus accumbens; RMTg, rostromedial tegmental nucleus; VTA, ventral tegmental area. Dashed lines indicate that there are no known direct connections.

The deleterious effects of ELS on reward circuits also involve alterations of innate stress neuromodulators such as CRF/CRFR1 and dynorphin (Dyn)/kappa opioid receptor (KOR) systems that contribute to the development of stress-induced drug seeking behaviors and negative affective states including anhedonia, social deficits and decreased motivation (as hallmark features of depression) following ELS (Land et al., 2008; Bruchas et al., 2010; Koob, 2010; Pautassi et al., 2012; Karkhanis et al., 2016; Mantsch et al., 2016; Bolton et al., 2018a; Knowland and Lim, 2018; Tejeda and Bonci, 2019). Recent work on the neuromodulatory regulation of LHb excitability and synaptic transmission by CRF/CRFR1 and Dyn/KOR signaling and their dysregulation by MD in male rats (Authement et al., 2018; Simmons et al., 2020) further highlight involvement of critical neuromodulators within LHb circuits that could underlie ELS-induced anhedonia, motivational deficits, drug seeking behaviors, and flexibility related behaviors. Intriguingly, LBN-induced anhedonia is also associated with high c-fos expression (indicative of increased neuronal activity) in the LHb and increased extrahypothalamic CRF neurotransmission from central amygdala (Bolton et al., 2018b), a brain region that also projects to the LHb (Hu et al., 2020). Therefore, additional insight into molecular mechanisms underlying CRF/CRFR1 and Dyn/KOR neuromodulation within LHb and its circuits in ELS models may offer novel therapeutic interventions with specificity for uncoupling these pathologically hyperactive stress signaling pathways following ELS (Figure 1B).

## Potential Insights into Lateral Habenula Function Utilizing More Complex, Ethologically Relevant Behaviors

Initial reports examining the role of the habenular complex, of which the LHb is a part, placed a wide range of behaviors from sexual functions, to circadian signaling under its control (Sutherland, 1982). As the toolkit to examine brain area contributions to behaviors has advanced, the range of behaviors typically associated with the LHb has narrowed to principally include aversive outcome signaling such as the omission of an expected reward, memory related functions described above, and adaptive behavioral selection such as during probabilistic reversal learning where reward contingencies in a T-maze are reversed once animals learn task contingencies (Nair et al., 2013; Baker et al., 2015; Sosa et al., 2021). Some of this is likely due to the ability to restrict interventions to spare fibers of passage or target specific cell identities. This no doubt ruled out contributions more likely to have come from nearby areas and the like. However, another likely contributor has been a reduction in the range of behavioral conditions examined due to limitations imposed by advanced recording and manipulation techniques.

What may have been lost with an increased focus on simplified behaviors is a greater appreciation for the complex ways a brain area can contribute to dynamic situations. Indeed, the prior two sections demonstrate that despite rapid advances in molecular and circuit understanding of the LHb, the nature of its contribution to integrating stress and memory related behaviors remains unclear. Recent work in the fear literature has demonstrated that ethologically relevant behaviors can reveal additional insight or even challenge long established roles for brain areas in behavior (Gross and Canteras, 2012; Gomez-Marin et al., 2014; Kim and Jung, 2018). Specifically, the inclusion of different scents, visual stimuli, or sounds may help more closely match what an animal experiences in the wild (Kim and Jung, 2018). For example, one such experiment involved placing rats in a continuous closed economy where food had to be accessed by risking shock during one period of the light dark cycle. Results revealed an amygdala dependent modulation of circadian rhythms can be elicited when the fear is timed to circadian cues (Pellman et al., 2015). In addition, realistic predator stimuli such as a plastic owl that surges from behind a hidden curtain while a hungry rat is foraging for food elicits opposite habituation to the fear related cues and willingness to enter a fear context in males and females than what is observed when using footshock and freezing as measures (Zambetti et al., 2019). Specifically, female rats are much less likely to approach the zone in which the owl surges than male rats.

When considering the role of the LHb in complex human psychiatric conditions, it is likely that similar additional insights into complex situations will also be gained by including ethological behavioral paradigms in animal models (Gomez-Marin et al., 2014) such as the closed economy, or simulated predator described above. Prior research utilizing ethologically relevant, complex behaviors have revealed a wealth of information into LHb contributions to decision-making. For example, Thornton and Evans (1982) observed that when rats were faced with an inescapable swimming scenario in the Morris water maze followed by a means of escape *via* rope climbing, habenula lesioned animals showed less flexible behavior (e.g., switching from trying to climb out *via* the edge of the pool to swimming to the middle to climb the rope) and a reduced likelihood of achieving escape. Combining such varied behaviors alongside the highly targeted molecular and physiological techniques now at the neuroscientist’s disposal may elaborate previously unknown, or recently forgotten roles for the LHb across a range of behaviors. For example, recent advances in behavioral tracking has led to the ability to precisely track positional information at a frame by frame granularity using automated behavioral coding (Nath et al., 2019; Nilsson et al., 2020). Understanding the neurophysiological changes associated with shifts in complex behaviors (such as during social interaction) and cue recognition could be critical to understanding how the LHb combines input from forebrain and memory related areas with stress related signals to influence downstream modulatory systems (Proulx et al., 2014).

To some extent, a revisiting of ethological behaviors in the LHb literature is already underway. Recent examples including realistic social aggression paradigms where mice are chronically socially defeated by a larger more aggressive strain (Flanigan et al., 2020), experiences of maternal deprivation during rearing described above (Shepard et al., 2018b), and social behavior in zebrafish examining decisions to fight or flee (Okamoto et al., 2021), are particularly relevant behaviors in the context of psychiatric conditions. In addition, when neural recordings have been obtained in freely behaving animals in dynamic environments, a much more complex picture of its role in ongoing behavior has emerged beyond signaling aversive stimuli (Baker et al., 2015; Lecca et al., 2020). Specifically, neural signals correlate strongly with velocity of animals as they seek rewards in open fields or in a T-maze (Sharp et al., 2006; Baker et al., 2015; Lecca et al., 2020). It is no doubt that behaviors such as conditioned place preference/avoidance, highly controlled delivery of aversive or appetitive stimuli, or sucrose preference have informed important theories of LHb function. Examining these theories within the broader view of the LHb in behavior summarized by Sutherland (1982), among others, will likely help clarify in what ways the conclusions from simplified paradigms contribute to more complex decision-making situations. For example, comparing results from effort based operant tasks such as progressive ratio (Zapata et al., 2017), with a more ethological behavior such as rats exerting effort to climb barriers (Sevigny et al., 2021) could help reveal the extent to which effort or fatigue is related to anatomical and physiological changes in LHb. This will further clarify potential LHb contributions to a wide range of psychiatric conditions.

## Summary/Conclusion

Over the past 20 or so years, there has been significant evolution in our understanding of the functional importance of the LHb. This has led to the generally-accepted views that the LHb plays a key role in associating context-dependent memory with one’s emotional state, and that dysfunction of this memory-emotion interface has neuropsychiatric consequences. As investigations continue to detail the dynamical nature of synaptic and circuit interactions of LHb function, it will be important to do so from a multi-level approach so that we will increasingly understand LHb function from its molecules to the circuits in which it is embedded. Having a more detailed comprehension of the LHb role in computing multimodal information regarding the emotional valence of a situation, prior stress experiences, and its contextual properties will likely help understanding some of the symptoms observed in pathologies associated with LHb dysfunctions such as depression (Li et al., 2013; Lecca et al., 2016; Nuno-Perez et al., 2018), addiction (Lecca et al., 2014; Velasquez et al., 2014; Mathis and Kenny, 2019) frontotemporal dementia (Bocchetta et al., 2016), and possibly schizophrenia (Zhang et al., 2017; Schafer et al., 2018).

The emergence of psychopathological symptoms is particularly striking when examined under stress induced contexts. A growing appreciation of the role of early life stress in LHb processing of emotional context is an example of the importance of understanding the complex interactions between memory and goal-directed behavior (Tchenio et al., 2017; Shepard et al., 2018b; Shepard and Nugent, 2021). Also, the yet unexplored LHb function in sleep appears relevant due to the relation between sleep and stress exposure (Goldstein and Walker, 2014; Vandekerckhove and Wang, 2018), and because sleep disturbances are key features of pathologies involving LHb dysfunction such as depression (Kudlow et al., 2013) and schizophrenia (Carruthers et al., 2021). Such studies examining the interaction between sleep and stress will likely bring new insights about cognitive deficits (memory loss, attention deficits, anhedonia) observed in depressive patients (Hammar and Ardal, 2009; Disner et al., 2011; Culpepper et al., 2017) and other populations with noted sleep disturbances.

Before we can develop efficient interventions to treat dysfunctions in memory, stress, sleep, and emotion processing, a number of questions remain to be addressed to further our understanding of the behavioral and neural mechanisms that underlie the LHb’s role in these contexts (Table 1). Overall, advances in our understanding of the functional significance of the LHb requires taking a multi-systems approach that includes the nature of the interactions between the LHb and its numerous afferent and efferent partners (Figure 1), as well as how the LHb plays central roles in many types of behaviors and types of memory. While behaviors including sleep, emotional processing, and decision-making often require the inclusion of more complex, or ethologically relevant behavioral assays, the insights gained from these studies will likely have important implications for understanding how observed cellular and circuit changes contribute to complex human psychopathologies.

**TABLE 1:** Outstanding issues and questions.

1.	What role does the LHb play in memory processing by healthy brains? To answer this question, we need to better understand the precise short- vs. long-term roles of the mPFC and hippocampus in LHb memory processing. Also enhancing the ecologically-relevance and complexity of the behavioral assessments should facilitate our ability to further dissect the role of the LHb in memory.
2.	What types of context memory information are conveyed to the LHb from the mPFC and hippocampus, and how is this information modified during stress or neuropsychiatric states such as depression and anxiety?
3.	Does the neural mechanism of communication between limbic cortex and the LHb change over time, and does this communication become altered in neuropsychiatric conditions?
4.	What are the cellular and network mechanisms by which the LHb integrates context memory (e.g., from mPFC and hippocampus) and motivational information related to emotional state? Future studies focused on the synaptic basis of memory formation and stress dysregulation of synaptic plasticity at these specific synaptic inputs to LHb are also warranted.
5.	It is well documented that sleep is essential for normal memory and emotion regulation. The strong anatomical connection between the suprachiasmatic nucleus and the LHb, and functional ties between the hippocampus and LHb, suggest that at least part of a memory influence of the LHb may be related to its processing during sleep. This is an understudied area of LHb function.
6.	LHb theories postulate that LHb output guides response flexibility and behavioral adaptation. The mechanisms involved in such behavioral guidance, however, are not clear, nor are the details by which stress might modify these output messages.
7.	Often memory disruption is thought to be the consequence of a disordered behavioral state such as that observed after stress. It is possible, however, that a memory disruption could lead to a stressed state. Distinguishing these interpretations is important yet challenging given the dynamic nature of neural systems.
8.	Are there differential impacts of maternal separation (predictable stress) and single maternal deprivation (unpredictable stress) when it comes to resistance or vulnerability to addictive behaviors, depression and other mood phenotypes?
9.	What are the critical neuromodulations within LHb that could underly anhedonia, motivational deficits, and drug seeking behaviors?

## Data Availability Statement

The original contributions presented in the study are included in the article/supplementary material, further inquiries can be directed to the corresponding author/s.

## Author Contributions

All authors contributed equally to this work and critically reviewed content and approved the final version of manuscript for submission.

## Author Disclaimer

The opinions and assertions contained herein are the private opinions of the authors and are not to be construed as official or reflecting the views of the Uniformed Services University of the Health Sciences or the Department of Defense or the Government of the United States.

## Conflict of Interest

The authors declare that the research was conducted in the absence of any commercial or financial relationships that could be construed as a potential conflict of interest.

## Publisher’s Note

All claims expressed in this article are solely those of the authors and do not necessarily represent those of their affiliated organizations, or those of the publisher, the editors and the reviewers. Any product that may be evaluated in this article, or claim that may be made by its manufacturer, is not guaranteed or endorsed by the publisher.
